# Literacy, but not memory, is associated with hippocampal connectivity in illiterate adults

**DOI:** 10.21203/rs.3.rs-3053775/v1

**Published:** 2023-06-16

**Authors:** Elisa de Paula França Resende, Vivian P. Lara, Ana Luisa C. Santiago, Clarisse V. Friedlaender, Howard J. Rosen, Jesse A. Brown, Yann Cobigo, Lênio L. G. Silva, Leonardo Cruz de Souza, Luciana Rincon, Lea T. Grinberg, Francisca I.P. Maciel, Paulo Caramelli

**Affiliations:** Universidade Federal de Minas Gerais; Faculdade de Ciências Médicas de Minas Gerais; Universidade Federal de Minas Gerais; Universidade Federal de Minas Gerais; University of California, San Francisco; University of California, San Francisco; University of California, San Francisco; Axial Inteligência Diagnóstica; Universidade Federal de Minas Gerais; Universidade Federal de Minas Gerais; University of California, San Francisco; Universidade Federal de Minas Gerais; Universidade Federal de Minas Gerais

**Keywords:** illiteracy, cognitive reserve, hippocampal connectivity, episodic memory

## Abstract

**Background:**

The influence of hippocampal connectivity on memory performance is well established in individuals with high educational attainment. However, the role of hippocampal connectivity in illiterate populations remains poorly understood.

**Methods:**

Thirty-five illiterate adults were administered a literacy assessment (Test of Functional Health Literacy in Adults - TOFHLA), structural and resting state functional MRI and an episodic memory test (Free and Cued Selective Reminding Test). Illiteracy was defined as a TOFHLA score below 53. We evaluated the correlation between hippocampal connectivity at rest and both free recall and literacy scores.

**Results:**

Participants were mostly female (57.1%) and Black (84.8%), with a median age of 50 years. The median TOFHLA literacy score was 28.0 [21.0;42.5] out of 100 points and the median free recall score was 30.0 [26.2;35] out of 48 points. The median gray matter volume of both the left and right hippocampi was 2.3 [2.1; 2.4] cm^3^. We observed a significant connectivity between both hippocampi and the precuneus and the ventral medial prefrontal cortex. Interestingly, the right hippocampal connectivity positively correlated with the literacy scores (β = 0.58, p = 0.008). There was no significant association between episodic memory and hippocampal connectivity. Neither memory nor literacy scores correlated with hippocampal gray matter volume.

**Conclusions:**

Low literacy levels correlate with hippocampal connectivity in illiterate adults. The lack of association with memory scores might be associated with low brain reserve in illiterate adults.

## Introduction

Life expectancy is increasing in low- and middle-income countries (LMIC), and consequently, the prevalence of dementia is rapidly rising. Between 2015 and 2050, the prevalence of dementia is expected to increase by 138% in countries like Brazil, compared to an increase of 56% in high-income countries (HIC) (Prince et al., 2015). Whereas most causes of dementia have no curative treatments so far, disease-modifying drugs for Alzheimer’s disease have a high cost and a controversial efficacy (Cummings et al., 2022). Moreover, co-pathologies in dementia are very common (Suemoto et al., 2017). Therefore, preventing dementia is a powerful strategy to mitigate the high burden of the disease on patients, caregivers, and society.

Research shows that in LMIC 48% of dementia cases could be prevented if 12 modifiable dementia-risk factors were controlled (Suemoto et al., 2022). Low educational level ranks highest among these factors. The prevalence and incidence of dementia in illiterate older adults is two and five times higher than in literate adults (César-Freitas et al., 2022; Nitrini et al., 2009; Ribeiro et al., 2022), respectively. Up to 19% of dementia cases can be attributed to low educational attainment in HIC and up to 30% in LMIC, where low education is more prevalent (Mukadam et al., 2019; Norton et al., 2014). An increase in educational attainment in HIC is believed to have contributed to the recently observed decline in dementia incidence(Wu et al., 2017). In the Framingham study, the incidence of dementia is declining only among persons who have at least a high-school degree (Satizabal et al., 2016). Although other socioeconomic determinants of health associated with high education may play a role in the apparent protective trends, several studies support education as an independent factor leading to lower risk of dementia (Lu et al., 2019; Sharp & Gatz, 2011; Wang et al., 2012).

Understanding how the illiterate brain process verbal and non-verbal cognitive tasks can help develop tailored strategies towards improving literacy skills to increase one’s memory abilities to mitigate the symptoms of dementia. Brain activation regarding letters and face recognition have different patterns in literate vs. illiterate individuals (Dehaene & Cohen, 2007; Dehaene et al., 2015; Dehaene et al., 2010). Additionally, regions of the brain involved in language processing have better white matter integrity in literate individuals compared to illiterates (Resende et al., 2017; Thiebaut de Schotten et al., 2014). However, it is unknown whether episodic memory correlates with hippocampal volumes and connectivity in illiterate individuals, classical neural substrates related to episodic memory in persons with high educational level.

Previous research in low literate older adults (mean of four years of formal education) showed that episodic memory correlated with the integrity of white matter bundles that connect the hippocampus with the precuneus and with hippocampal volume (Resende et al., 2017). However, this correlation was significant only amongst the group with more than four years of formal education (Resende, Rosen, et al., 2018). The role of hippocampal connectivity on episodic memory performance in adults is still a matter of debate (Aggleton & Brown, 1999; Bhattacharyya, 2017; Eichenbaum, 2004; Sidhu et al., 2013; van Kesteren et al., 2010) and there are no studies in illiterate adults. The default mode network is important for memory processing (Staffaroni et al., 2018) and it is affected in patients with dementia of the amnestic type (Malotaux et al., 2022; Seeley et al., 2009; Zhou et al., 2010).

In the present study, we used structural and functional MRI to define whether there was an association between episodic memory and hippocampal volumes and functional connectivity in illiterates. Understanding the brain mechanisms involved in episodic memory processing in illiterates can help unveil possible markers of successful interventions to improve memory in these populations, to mitigate the memory problems caused by neurodegenerative process that comes with aging.

## Methods

### Population

We used a community-based participatory research approach to collaborate with a basic-literacy training program for adults that is sponsored by the government. This program targets illiterate adults that did not have the opportunity to go to school when they were at the school age and want to learn how to read and write later in life. Adults aged 40 to 80 years-old that spontaneously enrolled in those late-life educational programs in the city of Belo Horizonte, Brazil, from February to July 2019 were invited to participate in the present research. Forty-three persons signed the informed consent and agreed to participate in the research. Sociodemographic and smoking habits were collected through a structured questionnaire. The level of physical activity was assessed with the Baecke scale (Baecke et al., 1982; Rocha et al., 1992). Depression, anxiety and alcohol abuse were investigated by the Mini International Neuropsychiatric Interview (Sheehan et al., 1998). All evaluations were conducted upon entry in the late-life literacy program before any literacy training. The socioeconomic levels were determined using the ABEPE (Brazilian Association of Research Companies) framework that categorize households into different socioeconomic levels. This classification considers various factors such as income, education, and ownership of goods to determine the living standards of households. The level A category represents the highest socioeconomic level with high income levels, advanced education, and ownership multiple properties and luxury goods. The level B category includes households with a relatively high socioeconomic status, although slightly lower than those in level A. These households generally have good incomes, tertiary education, and own properties and durable goods. The level C category encompasses households with a middle socioeconomic status. They usually have moderate incomes, secondary education, and may own a house or apartment. The levels D and E represent households with a lower socioeconomic status that often have low incomes, limited education, and may live in rented accommodations or informal settlements. They may face significant economic challenges and lack of access to basic services. They often live in poverty, struggling to meet their basic needs and relying on government assistance programs.

### Literacy and cognitive assessment

Participants that enroll in those late-life government sponsored programs have various degrees of reading and writing skills. Some never attended formal school while others attended for few years. Their reading abilities vary from inability to recognize letters to some reading capacity, without comprehending the meaning of the text. Therefore, we used the Test of Functional Health Literacy in Adults (TOFHLA) (Parker et al., 1995), validated for Brazilian Portuguese (Maragno et al., 2019), to evaluate the participant’s literacy skills across different levels. Previous studies determined that a score equal or lower than 53 defines illiteracy (Apolinario et al., 2015).

Global cognition was assessed by the Mini Mental State Examination (Brucki et al., 2003; Folstein et al., 1975) Episodic memory was assessed with the visual form (pictures) of the Free and Cued Selective Reminding test (FCSRT) (Grober et al., 2010; Zibetti et al., 2014). The FCSR-IR Free Recall sum-of-attempts was considered the proxy for episodic memory. Non-verbal intelligence was assessed by the Beta-3 test (Rabelo & Pacanaro, 2011), attention with the digit span test (de Paula et al., 2013), reading abilities with the Human Frontier Science Program reading test (Martins et al., 2023), words and sentence repetition with the Boston Diagnostic Aphasia Examination (Goodglass & Kaplan, 1983; Miotto et al., 2010) and verbal comprehension with the Token test (de Paula et al., 2013). Finally, participants performed the rapid naming of colors, letters, numbers, and objects(da Silva et al., 2020) and the Ekman’s facial emotion recognition test (Passarelli et al., 2018).

Global cognitive reserve was assessed with a structured questionnaire available in Portuguese, that includes years of education, leisure activities and occupational attainment (Nucci et al., 2012).

### Neuroimaging acquisition and analysis

Brain MRIs were acquired in a 3 Tesla Siemens Verio scanner with 3D-T1 and resting-state functional MRI (rsfMRI) acquisitions. The acquisition parameters were as follows. For 3D-T1: Field of View of 208×240×256 mm at reconstructed resolution of 1×1×1 mm, TE = min full echo, TR 2300 ms, TI 900 ms. For rsfMRI: Voxel resolution 2×2×2mm, Field of View of 220×220×163 mm, TE = 30 ms, TR 3000 ms, FA = 90°, time for acquisition 10 minutes.

Before any prepossessing of the images, all T1-weighted images were visually inspected for quality control. One image was excluded because of a large artifact. T1-weighted images undergone bias field correction using N3 algorithm, the segmentation was performed using SPM12 unified segmentation (Ashburner & Friston, 2005). A customized group template was generated from the segmented gray and white matter tissues and cerebrospinal fluid (CSF) by non-linear registration template generation using Large Deformation Diffeomorphic Metric Mapping framework (Ashburner & Friston, 2011). Native subjects’ space gray and white matter were geometrically normalized to the group template, modulated, and then smoothed in the group template. The applied smoothing used a Gaussian kernel with 8 ~ mm full width half maximum. Every step of the transformation was carefully inspected from the native space to the group template. For statistical purposes, linear and non-linear transformations between the group template space and International Consortium of Brain Mapping (ICBM) (Mazziotta et al., 1995) were applied. The Harvard-Oxford atlas (Desikan et al., 2006) was used to calculate the hippocampal volumes for each participant.

The rsfMRI analyses were done using the CONN (Whitfield-Gabrieli & Nieto-Castanon, 2012) release 20.b toolbox and SPM12 (Penny et al., 2011).

First, functional and anatomical data were preprocessed using a flexible preprocessing pipeline (Nieto-Castanon, 2020) including realignment with correction of susceptibility distortion interactions, slice timing correction, outlier detection, direct segmentation and MNI-space normalization, smoothing, and band-pass filtering. Functional data were realigned using SPM realign & unwarp procedure (Andersson et al., 2001), where all scans were coregistered to a reference image (first scan of the first session) using a least squares approach and a 6 parameter (rigid body) transformation (Friston et al., 1995), and resampled using b-spline interpolation to correct for motion and magnetic susceptibility interactions. Temporal misalignment between different slices of the functional data (acquired in interleaved Siemens order) was corrected following SPM slice-timing correction procedure (Henson et al., 1999; Sladky et al., 2011), using sinc temporal interpolation to resample each slice BOLD timeseries to a common mid-acquisition time. Potential outlier scans were identified using ART (Whitfield-Gabrieli, 2009) as acquisitions with framewise displacement above 0.9 mm or global BOLD signal changes above 5 standard deviations (Power et al., 2014). A reference BOLD image was computed for each subject by averaging all scans excluding outliers. Functional and anatomical data were normalized into standard MNI space, segmented into grey matter, white matter, and CSF tissue classes, and resampled to 2 mm isotropic voxels following a direct normalization procedure (Calhoun et al., 2017) using SPM unified segmentation and normalization algorithm (Ashburner & Friston, 2005) with the default IXI-549 tissue probability map template. Functional data were smoothed using spatial convolution with a Gaussian kernel of 8 mm full width half maximum. Last, BOLD signal timeseries were bandpass filtered between 0.01 Hz and 0.1 Hz.

In addition, functional data were denoised using a standard denoising pipeline(Friston et al., 1996) including the regression of potential confounding effects characterized by white matter timeseries (5 CompCor noise components), CSF timeseries (5 CompCor noise components), motion parameters and their first order derivatives (12 factors) (Friston et al., 1996), outlier scans (below 13 factors) (Power et al., 2014), session effects and their first order derivatives (2 factors), and linear trends (2 factors) within each functional run, followed by bandpass frequency filtering of the BOLD timeseries (Hallquist et al., 2013) between 0.008 Hz and 0.09 Hz. CompCor stands for Component-based noise correction method (Behzadi et al., 2007) that computes the average BOLD signal as well as the largest principal components orthogonal to the BOLD average, motion parameters, and outlier scans within each subject’s eroded segmentation masks. Those CompCor noise were estimated within the white matter and CSF.

Seed-based connectivity maps and ROI-to-ROI connectivity matrices were estimated characterizing the patterns of functional connectivity with 164 HPC-ICA networks (Whitfield-Gabrieli & Nieto-Castanon, 2012) and Harvard-Oxford atlas ROIs (Desikan et al., 2006). Functional connectivity strength was represented by Fisher-transformed bivariate correlation coefficients from a weighted general linear model (weighted-GLM (Nieto-Castanon, 2020)), defined separately for each pair of seed and target areas, modeling the association between their BOLD signal timeseries. To compensate for possible transient magnetization effects at the beginning of each run, individual scans were weighted by a step function convolved with an SPM canonical hemodynamic response function and rectified. The seed-based connectivity analyses were done placing a seed in each hippocampi using the Harvard-Oxford automated atlas (Desikan et al., 2006). The ROI-to-ROI connectivity matrices analyzed were the ones between each hippocampus and the ventral medial pre-frontal (VMPFC), each hippocampus (HC) and the Precuneus (PCC) and between the VMPFC and PCC.

Finally, the group-level analyses were performed using a GLM. For each individual voxel a separate GLM was estimated, with first-level connectivity measures at this voxel as dependent variables (one independent sample per subject), and groups as independent variables. Voxel-level hypotheses were evaluated using multivariate parametric statistics with random-effects across subjects and sample covariance estimation across multiple measurements. Inferences were performed at the level of individual clusters (groups of contiguous voxels). Cluster-level inferences were based on parametric statistics from Gaussian Random Field theory (Worsley et al., 1996). Results were thresholded using a combination of a cluster-forming p < 0.001 voxel-level threshold, and a familywise corrected p-FDR < 0.05 cluster-size threshold (Chumbley et al., 2010)

Demeaned age was used as a covariate in all neuroimaging analyses.

### Statistical analyses

Continuous variables were depicted in median and interquartile intervals; categorical variables were depicted in frequencies. GLM considering age, sex and total intracranial volume as covariates were used to calculate the correlation between episodic memory, literacy levels, brain connectivity and hippocampal volumes. In the first model, the FCSRT free-recall sum of attempts was the dependent variable, and the predictors were the functional connectivity between each HC separately and the VMPFC, between each HC and precuneus, and between the VMPFC and PCC, as well as with each hippocampal volume. In the second model, the literacy level measured by the TOFHLA total score was the dependent variable and the predictors were the same depicted above.

## Results

The final sample had 35 participants. We excluded three participants that had claustrophobia and did not tolerate the brain MRI, one participant whose scan had artifacts that precluded the analysis, three that were left-handed and one that scored 98 in the TOFLHA and was, therefore considered literate. The median age was 50 years, 57.1% (n = 20) of participants were women and 84.8% (n = 28) were Blacks (Table 1). The median TOFHLA score was 28 with an interquartile interval of 21.0 to 42.5.

**Figure 1:**
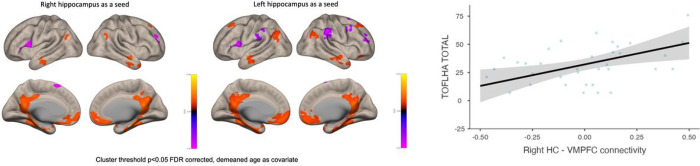
Correlation between hippocampal connectivity and low literacy levels. The statistical map is displayed on an inflated brain image. The heat maps represent the T statistical value for the connectivity between the right and left hippocampal seed and the other clusters. Blue means anticorrelation and red means positive correlation. The graph depicts the correlation between literacy levels measured by the Test of Functional Health Literacy Assessment (TOFHLA) and the right HC-VMPFC connectivity.

The seed-based connectivity analysis at rest showed a significant connectivity between both HC and the VMPFC and PCC, and other brain regions (Fig. 1). However, we failed to find a significant association between the HC-VMPFC connectivity and episodic memory measured by the FCSRT free recall sum of attempts (Table 2).

On the other hand, we found significant associations between the low TOFHLA scores and the HC-VMPFC connectivity (Table 3). Interestingly, the association was in opposite directions in each hippocampus. On the right side, the stronger the HC-VMPFC connectivity, the better the literacy scores (β = 0.58, p = 0.004), whilst on the left side, the stronger the connectivity, the worse the literacy scores (β=−0.39, p = 0.041).

Age and sex did not significantly correlate with the association between HC connectivity and memory or literacy scores.

## Discussion

In a group of middle-aged adults, the performance on a literacy test, even low enough to be considered illiterate per the literature (Apolinario et al., 2015), correlated with the HC-VMPFC connectivity. The association between low literacy levels and HC-VMPFC may suggest the role of even some literacy on cognitive reserve mechanisms. In contrast, we can speculate that the lack of association between episodic memory performance and hippocampal connectivity might reflect that this reserve is not enough to strengthen the role of hippocampal connectivity in memory abilities.

Cognitive reserve refers to distinct cognitive mechanisms, developed across the lifespan, that make a person more resilient or resistant to cognitive decline caused by brain damage (Stern et al., 2023). A higher level of cognitive reserve equips the brain to compensate through more efficient brain activation patterns that are more flexible and resilient to neurodegeneration or other forms of brain injury (Stern et al., 2023). Because higher cognitive reserve is associated with more tolerance to hippocampal atrophy (Murray et al., 2011), neurodegeneration (Hoenig et al., 2017; Wirth et al., 2014), and cerebrovascular (Fernandez-Cabello et al., 2016) pathologies, we believe that improving literacy levels might increase the HC-VMPFC connectivity and eventually prevent cognitive impairment in this population. Our finding may substantiate the hypothesis that improved hippocampal efficiency, reflected in stronger connections between the hippocampus and critical areas for memory processing such as the prefrontal cortex, may impact cognitive reserve even with some schooling. However, because our study was cross-sectional, we cannot demonstrate causality.

The TOFHLA test has been widely used to measure literacy level (Fan et al., 2021). Low literacy measured by the TOFHLA is associated with poor health outcomes (Apolinario et al., 2015; Fan et al., 2021). Although it is well established that the literate brain has different structural and functional properties (Dehaene & Cohen, 2007; Dehaene et al., 2015; Dehaene et al., 2010; Resende, Tovar-Moll, et al., 2018), the neural correlates of literacy measured by literacy tests, and not years of education, is less studied. A previous study showed that higher literacy skills measured by the REALM-SF test correlated with brain structural connectivity, but not with hippocampal volumes (Resende et al., 2022). Interestingly, we found that the very low literacy levels measured by the TOFHLA in our sample was significantly associated with the HC-VMPFC connectivity. We speculated that this finding may reflect how even low levels of literacy can relate to brain functioning, shedding light on a possible mechanism of cognitive reserve in this illiterate population.

In terms of episodic memory and brain connectivity, there is still a debate in the literature. The FCSRT is a traditional episodic memory test that has two versions (verbal and visual). The neural correlates of the verbal version have been more explored, while the visual version was less studied. Because the participants were illiterate, the visual version of the FCSRT was more appropriate. The few studies that explored the neural basis of the visual FCSRT test were conducted in persons with high educational level. One study with 14 participants compared the brain activation by the visual FCSRT between novel and repeated stimuli and showed that activations in left superior temporal and left prefrontal cortices were significantly associated with episodic memory (Diamond et al., 2007). Other brain areas activated through the FCSRT stimuli were the inferior parietal lobule, precuneus, hippocampus and parahippocampal gyrus (McLaren et al., 2012) as well as the posterior cingulate cortex and the precuneus connections (Edde et al., 2020).

In our study, the lack of association between episodic memory measured by the visual version of the FCSRT and the HC-VMPFC connectivity might be explained by the fact that we did not use task-based functional MRI as the previous studies used, but resting state functional MRI, which might be less sensitive to cognitive-brain correlations (Rasero et al., 2018). Another possibility is that illiterates use less their HC-VMPFC connectivity for memory processing, which might suggest a low cognitive reserve in this group. The fact that we found a significant relationship between literacy levels and the HC-VMPFC connectivity may support this theory, because, as the literacy levels increase, the association becomes stronger. In terms of structural neural correlates of the visual version of the FCSRT, the hippocampal volumes (Slachevsky et al., 2018) and brain areas involving visual processing (Arighi et al., 2018) have been implicated. The verbal version of the FCSRT, however, has been more studied. The hippocampal gray matter volume, mainly the left, has been consistently associated with the verbal version of FCSRT (Arighi et al., 2018; Epelbaum et al., 2018; Ezzati et al., 2016; Frank et al., 2022) in persons with high educational level. This association seems to be more evident in patients with AD (Novellino et al., 2018; Quenon et al., 2016; Sánchez-Benavides et al., 2010) and bvFTD (Bertoux et al., 2018; Poos et al., 2021) than in controls. The very low educational level of our sample combined with the lack of participants with dementia may explain why we did not find an association between episodic memory and hippocampal volumes. Indeed, two previous studies showed that the relationship between episodic memory and hippocampal volumes was moderated by educational level (O’Shea et al., 2018; Resende, Rosen, et al., 2018).

Our study has strengths and limitations. It is one of the first studies to look at the associations between the FCSRT visual version and hippocampal functional connectivity and gray matter volumes. The main limitation is the fact that it is cross sectional; therefore, not suitable for demonstrating causality. However, considering the scarcity of studies in illiterate adult populations, we consider it is an important first step into demonstrating whether late life literacy-training might have an impact on cognitive reserve. Nearly all current data available on the cognitive reserve field relate to formal education received in early life, but whether formal education provided during adulthood increases cognitive reserve with downstream benefits on dementia risk it is not known. Even considering the most recent drop in youth illiteracy due to LMIC efforts to provide formal education to school-age children, generations of adults remain illiterate and at higher risk of developing cognitive impairment later in life. If literacy-training in adulthood also improves cognitive reserve, even the current generation of low-educated adults could have benefits, an extremely important issue in LMIC where adult illiteracy rates often exceed 50% (Caribbean et al., 2022).

Our next goal is to explore the effects of adult-literacy training in brain structural and functional connectivity as well as in cognitive abilities, to determine whether adult-literacy acquisition might have a beneficial effect on dementia prevention. Eventually, we will be able to inform public policies to increase educational attainment in adulthood with a substantial impact on lowering dementia burden worldwide.

## Figures and Tables

**Table 1:** Participants characteristics

Characteristics n = 35	
Age (years)	50.0 [42.5; 58.0]
Sex female n(%)	20 (57.1%)
Self-reported race	
Blacks	28 (84.8%)
Whites	3 (9.1%)
Indigenous	2 (6.1%)
Unknown	2 (5.4%)
Socioeconomic level	
B	6 (17.1%)
C	11 (32.4%)
D-E	17 (50.0%)
Current anxiety	5 (14.3%)
Current depression	6 (17.1%)
Baecke physical exercise scale	3.0 [2.2; 5.8]
Cognitive Reserve Index	73.0 [70.0; 79.0]
MMSE	22.0 [21.0; 25.5]
Animals’ fluency/min	14.0 [12.0; 16.5]
Brief cognitive battery delayed recall	8.0 [7.5; 9.0]
TOFHLA total	28.0 [21; 42.5]
FCSRT free recall sum of attempts	30.0 [26.2; 35.0]
FCSRT cue efficiency	0.98 [0.96; 1.0]
FCSRT delayed free recall	11.0 [9.0; 13.0]
Word reading test	40.0 [0.0; 66.5]
Token verbal comprehension	27.0 [21.5; 29.0]
Rapid naming colors (seg)	45.5 [42.2; 57.7]
Rapid naming letters (seg)	41.5 [32.2; 54.2]
Rapid naming numbers (seg)	35.5 [31.2; 42.0]
Rapid naming objects (seg)	55.0 [47.2; 62.0]
Non-verbal intelligence Beta III test	6.0 [5.0; 7.7]
Right Hippocampal volume (mm3)	2.3 [2.1; 2.5]
Left Hippocampal volume (mm3)	2.3 [2.2; 2.4]

Values depicted in median and Interquartile interval.

See the text for more details about the socioeconomic levels.

**Table 2:** General linear models showing the association between FCSRT free recall sum-of-attempts scores and functional connectivity and hippocampal volume.

	β	t	p
Sex (Male)	0.09	0.23	0.822
Age	−0.38	−1.34	0.193
Right HC - VMPFC connectivity	0.15	0.63	0.535
Left HC - VMPFC connectivity	0.24	0.86	0.400
Right HC - PCC connectivity	−0.39	−1.67	0.109
Left HC - PCC connectivity	0.23	0.97	0.343
VMPFC - PCC connectivity	0.25	1.22	0.235
Left hippocampus volume	0.11	0.31	0.762
Right hippocampus volume	−0.37	−0.98	0.336
TIV	0.04	0.23	0.822

FCSRT: Free and Cued Selective Reminding Test, TIV: total intracranial volume, HC: hippocampus, VMPFC: Ventral medial pre-frontal cortex, PCC: precuneus

**Table 3:** General linear models showing the association between TOFHLA scores (literacy) and functional connectivity and hippocampal volume.

Names	β	t	p
Sex (Male)	0.25	0.65	0.522
Age	−0.16	−0.76	0.456
Right HC - VMPFC connectivity	0.58	2.90	0.008
Left HC - VMPFC connectivity	−0.35	−1.5	0.145
Right HC - PCC connectivity	0.10	0.51	0.616
Left HC - PCC connectivity	0.16	0.82	0.419
VMPFC - PCC connectivity	0.18	1.0	0.320
Left hippocampus volume	0.39	1.26	0.219
Right hippocampus volume	−0.35	−1.08	0.288
TIV	0.22	1.3	0.208

FCSRT: Free and Cued Selective Reminding Test, TIV: total intracranial volumes, HC: hippocampus, VMPFC: Ventral medial pre-frontal cortex, PCC: precuneus.

## Data Availability

The data that supports the findings are available upon reasonable request. Aggregated and anonymized data, as well as additional information related to the study methodology, can be made available to interested researchers. Requests for data access should be addressed to the corresponding author, Dr. Elisa de Paula França Resende (elisaresende@gbhi.org), who will assess each request on a case-by-case basis in consultation with the research team and in compliance with applicable data protection regulations and institutional policies.
